# Network Analysis for the Identification of Differentially Expressed Hub Genes Using Myogenin Knock-down Muscle Satellite Cells

**DOI:** 10.1371/journal.pone.0133597

**Published:** 2015-07-22

**Authors:** Adeel Malik, Eun Ju Lee, Arif Tasleem Jan, Sarafraz Ahmad, Kyung-Hyun Cho, Jihoe Kim, Inho Choi

**Affiliations:** 1 School of Biotechnology, Yeungnam University, Gyeongsan, Republic of Korea; 2 Perdana University Centre for Bioinformatics, MARDI Complex, Jalan MAEPS Perdana, 43400 Serdang, Selangor, Malaysia; 3 Bovine Genome Resources Bank, Yeungnam University, Gyeongsan, Republic of Korea; Ospedale Pediatrico Bambino Gesu', ITALY

## Abstract

Muscle, a multinucleate syncytium formed by the fusion of mononuclear myoblasts, arises from quiescent progenitors (satellite cells) via activation of muscle-specific transcription factors (MyoD, Myf5, myogenin: MYOG, *and* MRF4). Subsequent to a decline in Pax7, induction in the expression of MYOG is a hallmark of myoblasts that have entered the differentiation phase following cell cycle withdrawal. It is evident that MYOG function cannot be compensated by any other myogenic regulatory factors (MRFs). Despite a plethora of information available regarding MYOG, the mechanism by which MYOG regulates muscle cell differentiation has not yet been identified. Using an RNA-Seq approach, analysis of MYOG knock-down muscle satellite cells (MSCs) have shown that genes associated with cell cycle and division, DNA replication, and phosphate metabolism are differentially expressed. By constructing an interaction network of differentially expressed genes (DEGs) using GeneMANIA, cadherin-associated protein (CTNNA2) was identified as the main hub gene in the network with highest node degree. Four functional clusters (modules or communities) were identified in the network and the functional enrichment analysis revealed that genes included in these clusters significantly contribute to skeletal muscle development. To confirm this finding, *in vitro* studies revealed increased expression of CTNNA2 in MSCs on day 12 compared to day 10. Expression of CTNNA2 was decreased in MYOG knock-down cells. However, knocking down CTNNA2, which leads to increased expression of extracellular matrix (ECM) genes (type I collagen α1 and type I collagen α2) along with myostatin (MSTN), was not found significantly affecting the expression of MYOG in C2C12 cells. We therefore propose that MYOG exerts its regulatory effects by acting upstream of CTNNA2, which in turn regulates the differentiation of C2C12 cells via interaction with ECM genes. Taken together, these findings highlight a new mechanism by which MYOG interacts with CTNNA2 in order to promote myoblast differentiation.

## Introduction

Skeletal muscle, one of the most highly organized structures in the body, acts as a source of power for locomotion and other daily activities essential for survival. In vertebrates, development of skeletal muscle that commences at the embryonic stage ends only after postnatal growth during which an organism attains its fully developed size [1]. Skeletal muscle is unique in that this tissue arises from the fusion of mononuclear myoblasts accompanied by the expression of several myogenic regulatory factors (MRFs) following cell cycle exit to ensure the coordinated response to neural input [2–7]. Among basic helix-loop-helix (bHLH) and MADS-box families of MRFs (MYOD, MYF5, MRF4, and myogenin: MYOG) that play a critical role in myogenic differentiation, MYOD and MYF5 specifically play redundant roles during myoblast proliferation [8]. MYOG is responsible for terminal differentiation and cannot be compensated by other MRFs [9–12]. MYF5, MYOD, and MRF4 also spur the expression of genes that are essential for muscle satellite cells (MSCs) proliferation [8, 13, 14].

MSC progeny can be distinguished from their quiescent progenitors based on distinctive gene expression patterns. In adults, MSCs cycle through the steps of embryonic myogenesis to either add to or replace current muscle fibers [15–19]. Unlike the enigmatic status of genes that perform important functions in bovines, expression of a large number of genes (particularly those corresponding to different transcription factors) has been observed in mouse MSCs [20]. Therefore, it is important to delineate the expression profile of genes with unknown function in bovine-derived MSCs. Our interests in obtaining the regulatory profile of genes with important functions in mouse MSCs led us to perform the current investigation with bovine MSCs to have a clear understanding of bovine muscle development. By employing microarray, expressed sequence tag (EST) followed by RNA-Seq techniques to MSCs satellite cell analysis, we were able to delineate the regulatory network of genes corresponding to different transcription factors and certain prominent members of the extracellular protein family, involved in controlling myoblast differentiation [20, 21]. We were able to elucidate and assign specific roles to certain genes, such as transthyretin, that are novel with respect to their involvement in myogenesis [22]. While investigating the gene expression profile of MYOG knock-down (MYOG_kd_) in bovine MSCs using RNA-Seq, we observed differential expression patterns of many genes, particularly those involved in biological pathways such as cell proliferation and DNA replication (up-regulated) or phosphate metabolic processes (down-regulated) [23].

A large number of high-throughput studies have been performed to explore the functional roles of genes found to have altered expression patterns during skeletal muscle differentiation [5, 24–26]. Microarray [12, 27, 28] along with RNA-Seq [29] studies have improved our knowledge of myogenesis by identifying diverse types of target genes encoding myogenic transcription factors or novel myogenic regulatory factors which govern the fusion of myoblasts into myotubes that were otherwise hard to detect with conventional methods. In spite of these improvements, the roles that these genes play in skeletal muscle development as well as the understanding of underlying molecular mechanisms are still poorly understood. Therefore, we performed the current study to investigate the molecular mechanism underlying muscle cell differentiation by identifying genes involved in myogenesis. We created an interaction network of differentially expressed genes (DEGs) as well as additional related genes predicted by GeneMANIA, identified hub genes in the network based on their node degree distribution, and performed a functional study using gene knock-down. Furthermore, we identified functional modules in this network of DEGs, and an enrichment analysis of these modules was carried out by the Database for Annotation, Visualization and Integrated Discovery (DAVID) functional analysis tool. Our findings offer new insights into the role of hub genes in skeletal muscle development that will help to develop strategies for improving the meat quality and in combating muscle diseases.

## Materials and Methods

### Datasets and network analysis

For this study, 230 up- and 223 down-regulated genes identified with bovine MYOG_kd_ RNA-Seq data were analyzed [23]. Functional interactions between these DEGs were predicted by the GeneMANIA webserver [30]. In addition to the DEGs, 50 additional genes were used to create the interaction network using the Gene Ontology (GO) term “biological process” and *Homo sapiens* as a source species. Three interaction networks were created using up-regulated genes, down-regulated genes, and a combination of the up- and down-regulated genes. Relationships of genes in the network in terms of coexpression, physical and genetic interactions, pathways, colocalization, protein domain similarity, and predicted interactions were evaluated.

### Detection of hub genes

In scale-free biological networks [31], nodes having large number of interacting partners represent hubs in the network. Hubs were detected by calculating the node degree distribution [32] using the Network Analyzer (http://apps.cytoscape.org/apps/networkanalyzer) plugin of Cytoscape.

### Community analysis and comparison of different clustering methods

The three interaction networks were visualized with Cytoscape 2.8.2 [33]. We also compared three widely used network clustering algorithms including MCL [34, 35], MCODE [36], and the greedy algorithm (GLay) [37] using clusterMaker [38] and GLay plugins [39]. To explore the enriched biological functions within each cluster, the detected clusters were subjected to functional enrichment analyses by the DAVID tool (http://david.abcc.ncifcrf.gov/home.jsp). For the enrichment analysis, only communities with at least 10 nodes were evaluated.

### Bovine MSCs culture

Bovine skeletal muscles were collected from the hind leg of 24- to 26-month old Korean cattle [40] with a body weight of 550–600 ㎏. The animals were handled according to a protocol approved by the Animal Care and Concern Committee of the National Institute of Animal Science (Republic of Korea). The collected muscle tissue was minced and digested with trypsin-EDTA (Gibco, CA, USA), centrifuged at 90 × g for 3 min, and the upper phase was passed through a 40-㎛ cell strainer (Falcon, NY, USA). Following centrifugation of the filtrate at 2,500 rpm, the resulting pellet was collected, washed with Dulbecco’s modified Eagle’s medium (DMEM; HyClone Laboratories, UT, USA), and cultured at 37°C under 5% CO_2_ in DMEM supplemented with 10% fetal bovine serum (FBS; HyClone Laboratories) and 1% penicillin/streptomycin (P/S). The culture medium was changed every other day. To induce differentiation, the cells were allowed to grow without reducing serum (DMEM with 10% FBS and 1% P/S) for 10, 12, 14, and 16 day. Bovine MSCs used in this study were provided by the Bovine Genome Resources Bank of Yeungnam University (Gyeongsan, Republic of Korea).

### shRNA construction and MYOG knock-down in bovine MSCs

Bovine MYOG shRNA was designed using nucleotide sequence information obtained from National Center for Biotechnology Information (NCBI; AB257560) and cloned with a pRNAT-U6.2/Lenti vector (GeneScript, NJ, USA). MYOG shRNA or scrambled vector (MYOG_wt_) were transfected in 293 FT cells using CaCl_2_ to generate viral particles. After 2 days of transfection, the supernatant containing viral particles expressing shRNA specific for bovine MYOG or the scrambled vector was collected and used to transfect MSCs (Day 8). The transfected cells were selected with 50 ㎍/㎖ of G418 (Calbiochem, CA, USA). The selected cells were allowed to differentiate and harvested at Day 21. The following oligonucleotide was used to generate MYOG shRNA: 5′- GGATCCCGCGCAGACTCAAGCCGCCGGTGTTCAAGAGACACCTTCTTGAGTCTGCGCTTTTCCAACTCHGAG-3′ (sense). 293 FT cells were procured from Korea University Dr. Chun’s lab, Republic of Korea.

### C2C12 cell culture

Mouse C2C12 cells (kindly provided by the Korean Cell Line Bank, Republic of Korea) were cultured in DMEM (HyClone Laboratories) supplemented with 10% FBS (HyClone Laboratories) and 1% P/S (Invitrogen, CA, USA) at 37°C in 5% CO_2_. For differentiation, cells grown to 70% confluence were incubated with differentiation media (DMEM with 2% FBS), and cultured for 0, 2, 4, and 6 days during which time the medium was changed every 2 day.

### MYOG and CTNNA2 knock-down in C2C12 cells

MYOG expression was knocked down using shRNA. C2C12 cells grown to 30% confluence were transfected with 1 ng of vector containing MYOG shRNA or the scrambled vector using transfection reagent (Santa Cruz Biotechnology, CA, USA). Transfected cells treated with 2 ㎍/㎖/ puromycin (Santa Cruz Biotechnology) for selection were grown to 70% confluence before switching to differentiation media. MYOG knock-down cells were cultured in differentiation media. Detailed information describing the shRNA sequences is provided in **A) in S1 Table.**

For CTNNA2 knock-down using siRNA, C2C12 cells grown to 50% confluence were transfected with 100 μM of control or CTNNA2-specific siRNA (Thermo, Waltham, CO, USA) using Lipofectamine (Invitrogen) in transfection medium (Gibco, Grand Island, NY, USA). The transfected cells were grown to 70% confluence before switching to differentiation media. CTNNA2 knock-down cells were cultured in differentiation media. Detailed information describing the siRNA sequences is provided in **B) in S1 Table.**

### RNA extraction and real-time RT-PCR

Cells were harvested with TRIzol reagent (Invitrogen) according to the manufacturer’s protocol. Total RNA was extracted and stored in diethylpyrocarbonate (Sigma Aldrich)-treated H_2_O at -80°C until it was used for further experiments. Complementary DNA (cDNA) was synthesized from 1 ㎍ of total RNA using reverse transcriptase (Invitrogen, Carlsbad, CA). Reverse transcription was then performed at 42°C for 50 min and 72°C for 15 min. Subsequently, 2 ㎕ of cDNA product and 10 pmol each of gene-specific primer were used for PCR with a 7500 real-time PCR system (Applied Biosystems, Foster City, CA, USA). A Power SYBR Green PCR Master Mix (Applied Biosystems) was used as the fluorescence source. Primers were designed with Primer 3 software (http://frodo.wi.mit.edu) using sequence information obtained from the NCBI. The primer sequences are provided in **S2 Table.**

### Immunocytochemistry

C2C12 cells grown in a covered glass-bottom dish (SPL, Yeoju, Republic of Korea) were treated with differentiation medium and stained for CTNNA2 protein following 0 and 6 days of incubation. Briefly, the cells were rinsed with PBS and fixed with 4% formaldehyde. Following permeabilization with 0.2% Triton X-100 (Sigma-Aldrich, MO, USA) for 5 min, the cells were incubated overnight with anti-CTNNA2 antibody (1:50; Abcam, MA, USA) at 4°C in a humid environment. Secondary antibody (1:100; Alexa Fluor 488 goat anti-rabbit SFX kit; Invitrogen) was then applied for 1 h at room temperature. Next, the samples were rinsed with PBS after which the nuclei were counterstained with 4’ 6’-diamino-2-phenylindole (DAPI; Sigma-Aldrich). Finally, pictures were taken using a fluorescent microscope equipped with a digital camera (Nikon, NY, USA).

### Fusion index determination

Cell nuclei were stained with Giemsa G250 (Sigma-Aldrich) and pictures were captured randomly in three different fields using digital camera equipped with microscope. The number of nuclei in myotubes and total number of nuclei in the cells were also counted in each field. The fusion index was calculated as the percentage of nuclei incorporated into myotubes versus the total number of nuclei.

### Western blot analysis

After washing with ice-cold PBS, cells were lysed with RIPA buffer (Thermo) containing protease inhibitor cocktail (Thermo). Total protein was isolated by centrifugation of the lysate at 12,000 rpm for 10 min at 4°C after which the protein concentration was determined by the Bradford method. The total protein (40 ㎍) was separated in 8% or 10% SDS-polyacrylamide gels by electrophoresis and transferred to PVDF membranes (Millipore, MA, USA). The blots were subsequently blocked with 3% skim milk (BD, MD, USA) or BSA (Sigma-Aldrich) in TBST for 1 h and then incubated overnight at 4°C with antibodies against CTNNA2 (1:500, Santa Cruz Biotechnology), type I collagen α1 (COL1 α 1; 1:2500, Abcam), Myostatin (MSTN; 1:400, Santa Cruz Biotechnology), or β-actin (1:2000, Santa Cruz Biotechnology) diluted with 1% skim milk or BSA in TBS. After washing with TBST, the blots were incubated with horseradish peroxidase (HRP)-conjugated secondary antibody (goat anti rabbit or mouse, Santa Cruz Biotechnology) for 1 h at room temperature. After washing with TBST, antibody binding was detected with Super Signal West Pico Chemiluminescent Substrate (Thermo).

### Statistical analysis

Mean values for normalized expression were compared using Tukey’s studentized range (HSD) to identify significant differences in gene expression. P-values < 0.05 were considered statistically significant. Real-time RT-PCR data were analyzed with a one-way ANOVA using PROC GLM in the SAS package ver. 9.0 (SAS Institute, Cary, NC, USA).

## Results

### DEGs and network construction

**Fig 1:**
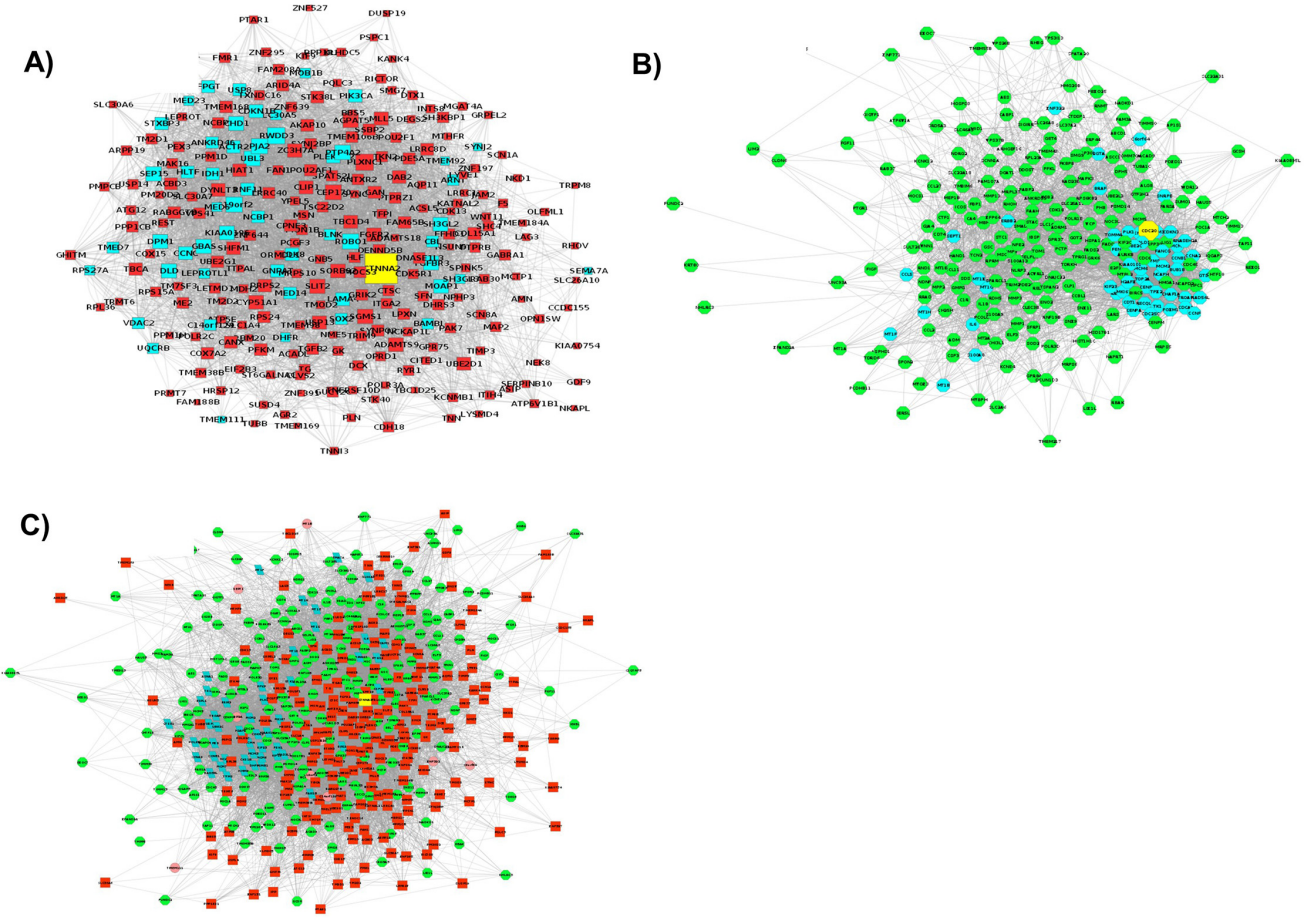
The interaction networks for DEGs as predicted by GeneMania and visualized in Cytoscape. The three networks represent interactions for A) down-regulated genes, B) up-regulated genes, and C) a combination of up- and down-regulated genes.

A total of 230 up-regulated and 223 down-regulated genes identified by comparing MYOG_wt_ and MYOG_kd_ primary bovine MSCs were studied [23]. The GeneMANIA webserver was used to predict interactions between the DEGs and 50 additional related genes in the network using the GO term “biological process” and source organism *Homo sapiens* as additional parameters. Out of the 230 up- and 223 down-regulated genes, GeneMANIA was able to recognize 217 and 215 genes, respectively. Using these DEGs and the 50 related genes identified by GeneMANIA, three interaction networks were created. The first interaction network (***Network 1***) was created using 215 down-regulated genes and showed enrichment of processes related to the TGF-β signaling pathway (**S3 Table**). In this network, red squares represented down-regulated genes and nodes shown in cyan were GeneMANIA-predicted genes (**Fig 1A**). The second network (***Network*** 2) contained 217 up-regulated genes (nodes in green) and GeneMANIA-predicted genes (nodes in cyan; **Fig 1B**). Processes related to the cell cycle were the main processes in this network (**S4 Table**). The final network (***Network 3***) consisted of 432 genes (both up- and down-regulated) as well as GeneMANIA-predicted genes (**Fig 1C**). The three interaction networks predicted by GeneMANIA were exported to Cytoscape for filtering (to remove duplicate edges and self-loops), and topological properties such as node degree were assessed. After removing the duplicate edges and self-loops, ***Network 1*** contained 264 nodes and 4545 edges whereas ***Network 2*** had 264 nodes and 4309 edges. Similarly, the filtered ***Network 3*** (the main network in this study) had 481 nodes and 11374 edges, and functions related to the cell cycle were overrepresented (**Table 1**).

**Table 1:** Functional enrichment of differentially expressed genes (DEG) and 50 related genes in the network as reported by GeneMANIA.

Functions	FDR
response to zinc ion	0.0000142
regulation of growth	0.000176
negative regulation of growth	0.000315
DNA strand elongation involved in DNA replication	0.000315
response to cadmium ion	0.000315
MCM complex	0.000315
interphase of mitotic cell cycle	0.00035
interphase	0.000386
S phase of mitotic cell cycle	0.000386
DNA strand elongation	0.000389
mitosis	0.000389
nuclear division	0.000389
cell cycle checkpoint	0.000447
regulation of cell cycle process	0.000477
S phase	0.000477
M phase of mitotic cell cycle	0.000642
G1/S transition of mitotic cell cycle	0.00088
organelle fission	0.000981
cellular response to metal ion	0.00108
cellular response to inorganic substance	0.00153
regulation of mitotic cell cycle	0.00163
zinc ion binding	0.00163
perinuclear region of cytoplasm	0.00202
regulation of transcription involved in G1/S phase of mitotic cell cycle	0.00711
DNA-dependent DNA replication	0.0105
regulation of peptidase activity	0.0127
cell growth	0.0129
M/G1 transition of mitotic cell cycle	0.0169
microtubule associated complex	0.0204
response to interleukin-6	0.0205
transition metal ion binding	0.0211
regulation of endopeptidase activity	0.0231
enzyme inhibitor activity	0.0281
DNA replication	0.029
regulation of proteasomal protein catabolic process	0.0322
acute inflammatory response	0.0407
leukocyte migration	0.047
positive regulation of cell cycle process	0.0509
regulation of protein catabolic process	0.0522
regulation of cell growth	0.0553
proteasomal protein catabolic process	0.0618
positive regulation of cell cycle	0.0727
leukocyte chemotaxis	0.0734
acute-phase response	0.0734
neutrophil chemotaxis	0.0734
regulation of mitosis	0.0786
regulation of nuclear division	0.0786
regulation of proteolysis	0.082
cellular response to drug	0.082
anaphase-promoting complex-dependent proteasomal ubiquitin-dependent protein catabolic process	0.082
endopeptidase regulator activity	0.083
regulation of leukocyte migration	0.0896
proteasomal ubiquitin-dependent protein catabolic process	0.0938
cell chemotaxis	0.0965
microtubule-based process	0.0965
mitotic prometaphase	0.0965

### Identification of hub genes

**Fig 2:**
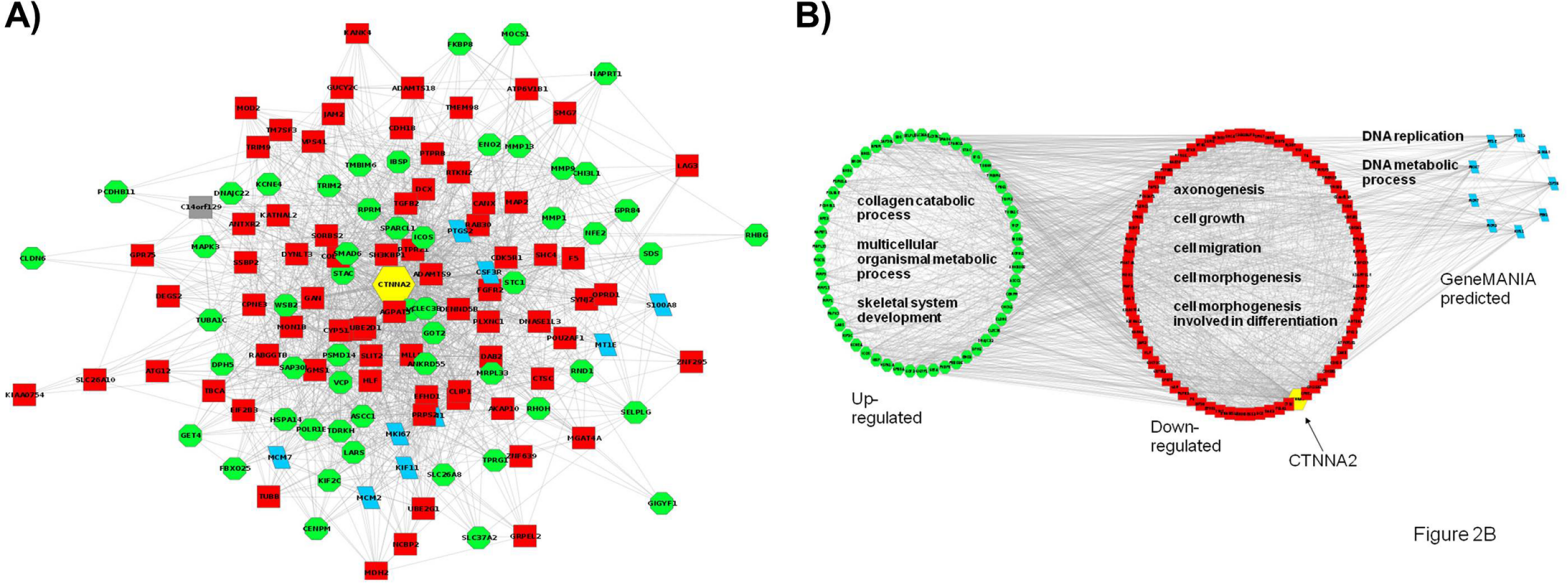
CTNNA2 is a hub in the network of down-regulated genes as well as the network created by combining the up- and down-regulated genes. **A)** The hub CTNNA2 (yellow) and its first neighbors. Genes that were up-regulated in MYOG_kd_ cells are shown in green whereas the down-regulated genes are shown in red. The genes predicted by GeneMania are shown in cyan. **B)** Functional enrichment of first neighbors of CTNNA2. The green circles represent up-regulated gene nodes and are enriched in metabolic processes. The larger red circles represent down-regulated nodes and are enriched in processes related to cell growth, morphogenesis, and migration. The smallest circle (cyan) represents the group of genes predicted by GeneMania and is enriched for DNA replication and DNA metabolic processes.

In order to identify potential hub genes, the node degree of each node in all three networks was calculated with the Network-Analyzer plugin of Cytoscape. Genes with the highest node degree were considered hub genes in each of these networks. CTNNA2 was the hub gene in ***Network 1*** and ***Network 3*** with node degrees of 89 and 136, respectively. In ***Network 2***, CDC20 was the top hub with a node degree of 83. In **Table 2**, all genes that had a node degree ≥100 in ***Network 3*** are listed. Since CTNNA2 was identified as the top hub gene in two out of three interaction networks (***Networks 1*** and ***3***), *in vitro* studies were designed to explore the functional significance of this gene in skeletal muscle development using primary bovine MSCs. The top hub gene CTNNA2 and its interacting partners are presented in **Fig 2A** In **Fig 2B**, it can be observed that these interacting partners are involved in collagen catabolic processes and skeletal muscle development (up-regulated genes), or processes related to cell growth and morphogenesis (down-regulated genes). Similarly, the GeneMANIA-predicted first neighbors of CTNNA2 were involved in either DNA replication or DNA metabolic processes (**Table 3**).

**Table 2:** Summary of 13 hub genes detected by network analysis.

Gene ID	Uniprot ID	Protein Name	GO term (Biological Process)
CTNNA2	A6QPC5	Catenin alpha-2	cell adhesion
GOT2	P12344	Aspartate aminotransferase, mitochondrial	cellular amino acid metabolic process, biosynthetic process
PLXNC1	E1BDY7	Plexin-C1	signal transduction, multicellular organismal development
RPRM	Q1RMT2	Protein reprimo	cell cycle arrest, regulation of mitotic cell cycle
MKI67	P46013	MKI67 FHA domain-interacting nucleolar phosphoprotein	DNA metabolic process, cell proliferation
ADAMTS9	E1BI72	A disintegrin and metalloproteinase with thrombospondin motifs 9	proteolysis
FEN1	Q58DH8	Flap endonuclease 1	DNA repair
CDC6	Q99741	Cell division control protein 6 homolog	DNA replication
KIF11	E1BF29	Kinesin-like protein	microtubule-based movement
STAC	A0JNJ1	SH3 and cysteine-rich domain-containing protein	intracellular signal transduction
PLK1	Q2TA25	Serine/threonine-protein kinase PLK1	protein phosphorylation
MCM3	A4FUD9	DNA replication licensing factor MCM3	DNA replication, DNA replication initiation
MAD2L1	Q13257	Mitotic spindle assembly checkpoint protein MAD2A	mitotic spindle assembly checkpoint

**Table 3:** Top enriched GO terms for CTNNA2 and its first neighbors.

Term	P-Value
GO:0048666~neuron development	0.007508112
GO:0030182~neuron differentiation	0.011068049
GO:0030574~collagen catabolic process	0.011911391
GO:0007049~cell cycle	0.012624252
GO:0009057~macromolecule catabolic process	0.013262195
GO:0016049~cell growth	0.014377241
GO:0001503~ossification	0.015704029
GO:0000904~cell morphogenesis involved in differentiation	0.016507381
GO:0060348~bone development	0.019609996
GO:0044243~multicellular organismal catabolic process	0.019723571
GO:0022402~cell cycle process	0.019741599
GO:0031175~neuron projection development	0.020394638
GO:0032963~collagen metabolic process	0.022693643
GO:0044259~multicellular organismal macromolecule metabolic process	0.027468976
GO:0016337~cell-cell adhesion	0.028182696
GO:0016477~cell migration	0.028182696
GO:0016477~cell migration	0.028182696
GO:0008361~regulation of cell size	0.029200653
GO:0007155~cell adhesion	0.031998038
GO:0022610~biological adhesion	0.032284455

### Community analysis

**Fig 3:**
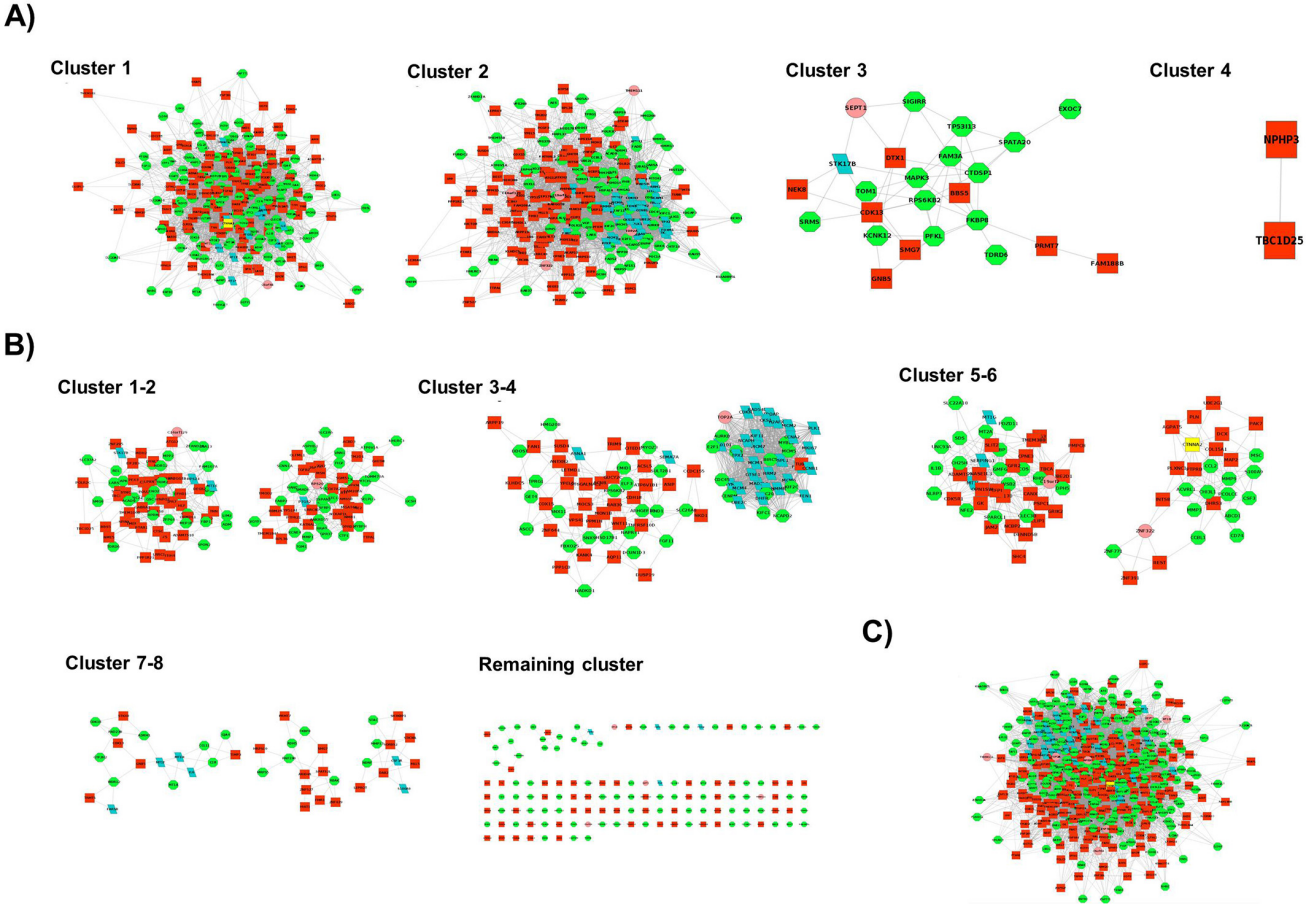
Communities generated by three different clustering methods. **A)** Cluster 1–4: Greedy algorithm (GLay)-generated communities are shown. In each community, up-regulated nodes are represented by green circles whereas the down-regulated nodes are shown in red. As presented in the figure, most nodes in a network were assigned to a community. Community 1 has CTNNA2 as the hub node highlighted in yellow. **B)** Cluster 1–8, along with remaining one: MCODE-generated communities are shown. Many nodes are not clustered, and the top hub CTNNA2 is grouped into community 6. **C)** MCL produced only one large community with more than 10 nodes.

Since ***Network 3*** was largest of the three networks and contained both up- and down-regulated genes, we selected this network to identify functional modules using greedy algorithm (GLay). A total of four functional clusters were detected with only the first three clusters having more than ten genes; these were further subjected to functional analysis to identify enriched GO terms. **Fig 3A (Cluster 1–4)** shows the functional modules detected by GLay. A DAVID functional analysis tool was employed to categorize the genes in each cluster and observe the overrepresented GO terms in all three modules (**Table 4**). Overall, 233 enriched GO terms were identified in all three clusters. One hundred and thirty-six statistically significant (p-value ≤ 0.05) GO terms were overrepresented in cluster 1, which was the largest detected cluster. Among the 10 most significant enriched factors, processes related to homeostasis were more prominent in cluster 1 (**Table 4A and S5 Table**). Other overrepresented processes in this cluster include leukocyte migration, response to wounding, and cell migration and motion. A total of 93 GO terms that were enriched in cluster 2 included processes related to the cell cycle and DNA replication (**Table 4B and S6 Table**). Cluster 3 was the smallest of all the modules detected. It consisted of only four statistically significant phosphorous metabolism-related GO terms such as protein amino acid phosphorylation and the phosphorous metabolic process (**Table 4C**). No enriched category was observed in cluster 4 as this cluster was omitted from the functional analysis because it only contained two genes.

**Table 4:** Enriched GO terms in each cluster detected by GLay.

Term	P-value
**A) Cluster 1**	
GO:0050900~leukocyte migration	1.316E-05
GO:0009611~response to wounding	1.5127E-05
GO:0055080~cation homeostasis	4.6482E-05
GO:0050801~ion homeostasis	5.6871E-05
GO:0006873~cellular ion homeostasis	6.7452E-05
GO:0055082~cellular chemical homeostasis	8.1336E-05
GO:0006928~cell motion	0.00011197
GO:0016477~cell migration	0.00012824
GO:0055066~di-, tri-valent inorganic cation homeostasis	0.00012879
GO:0030003~cellular cation homeostasis	0.00022731
**B) Cluster 2**	
GO:0007049~cell cycle	2.611E-15
GO:0000278~mitotic cell cycle	1.6135E-13
GO:0022403~cell cycle phase	2.3341E-12
GO:0022402~cell cycle process	4.307E-12
GO:0007067~mitosis	4.6172E-11
GO:0000280~nuclear division	4.6172E-11
GO:0000087~M phase of mitotic cell cycle	6.3176E-11
GO:0048285~organelle fission	9.2574E-11
GO:0000279~M phase	1.8784E-10
GO:0006260~DNA replication	2.4533E-09
**C) Cluster 3**	
GO:0006468~protein amino acid phosphorylation	0.00187298
GO:0016310~phosphorylation	0.00414241
GO:0006793~phosphorus metabolic process	0.00947152
GO:0006796~phosphate metabolic process	0.00947152

**Fig 4:**
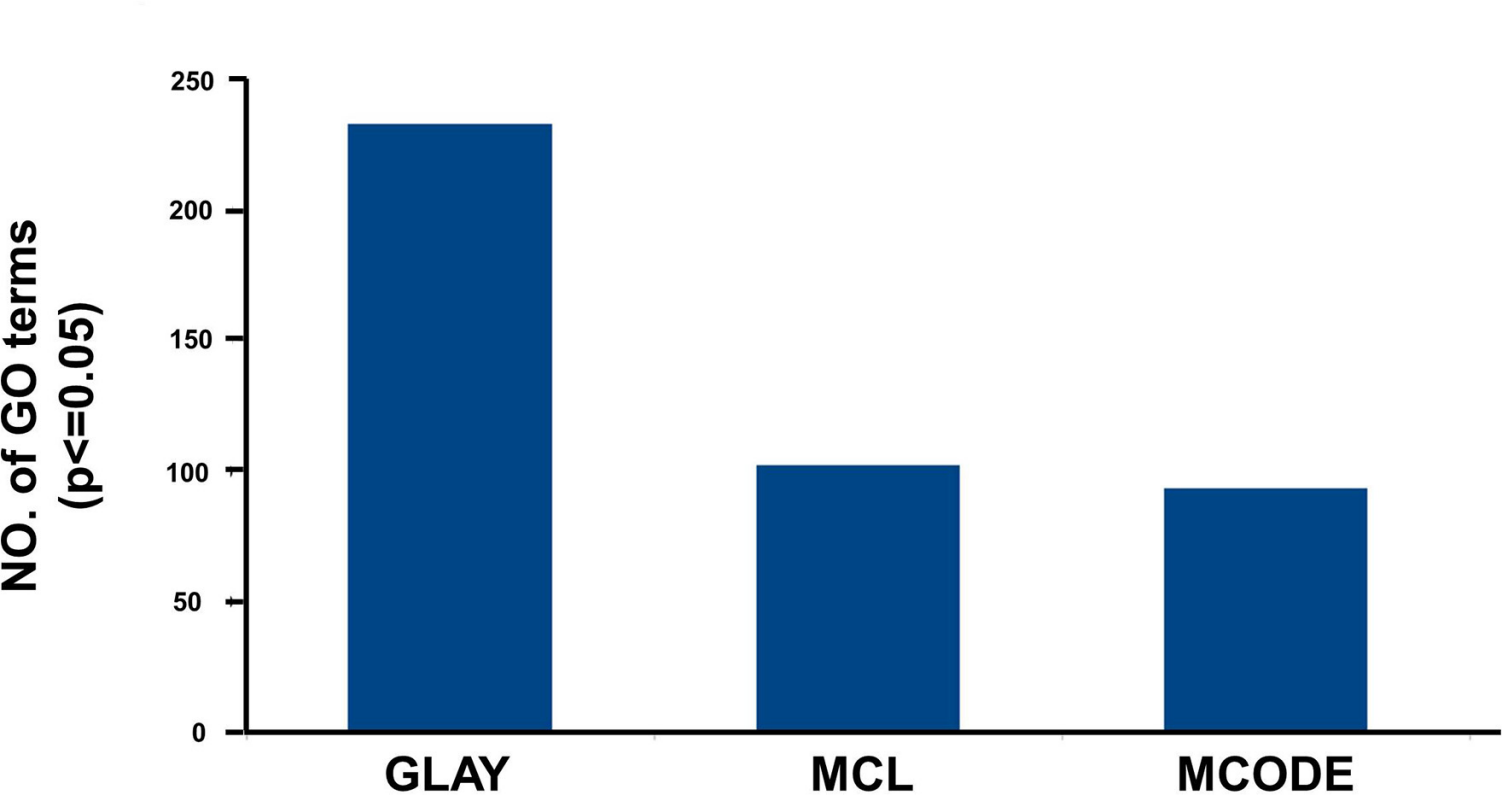
Comparison of statistically significant GO terms reported by different algorithms. In the figure it can be observed that GLay produced the maximum number of statistically significant GO terms.

In addition to GLay, we also compared the cluster analysis results with two other well-known clustering programs: MCODE and MCL. MCODE detected 12 clusters, but only nine that contained ≥ 10 nodes were selected for enrichment analysis (**Fig 3B Cluster 1–8 along with remaining one**). These nine modules were associated with 93 statistically significant GO terms. Clusters 1, 8, and 9 did not have any enriched categories. Cluster 2 was enriched in leukocyte migration and enzyme-linked receptor protein signaling pathway, whereas cluster 3 showed overrepresentation of biosynthetic processes such as those involved in nitrogen compound and cofactor biosynthesis. Cluster 4, the largest cluster detected by MCODE, included processes related to the cell cycle and cell division. Cluster 5 was mainly associated with regulation processes that govern nucleocytoplasmic and intracellular transport, response to stimuli, immune effector activity, and neuron differentiation. In cluster 6, the enriched terms included cell motility, cell motion, and phosphorous metabolic process whereas cluster 7 was associated with responses to wounding and inflammation in addition to the phosphorous metabolic process (**A) in S7 Table**). On the other hand, a total of seven clusters were detected by MCL with only one large cluster having more than 10 nodes (**Fig 3C**). This cluster had 102 significantly enriched GO terms with the most enriched representing processes related to the cell cycle and DNA replication (**B) in S7 Table**). For comparison, the numbers of statistically significant GO terms reported by three different algorithms are summarized in **Fig 4**.

### CTNNA2 expression during differentiation and effects of MYOG knock-down in bovine MSCs

**Fig 5:**
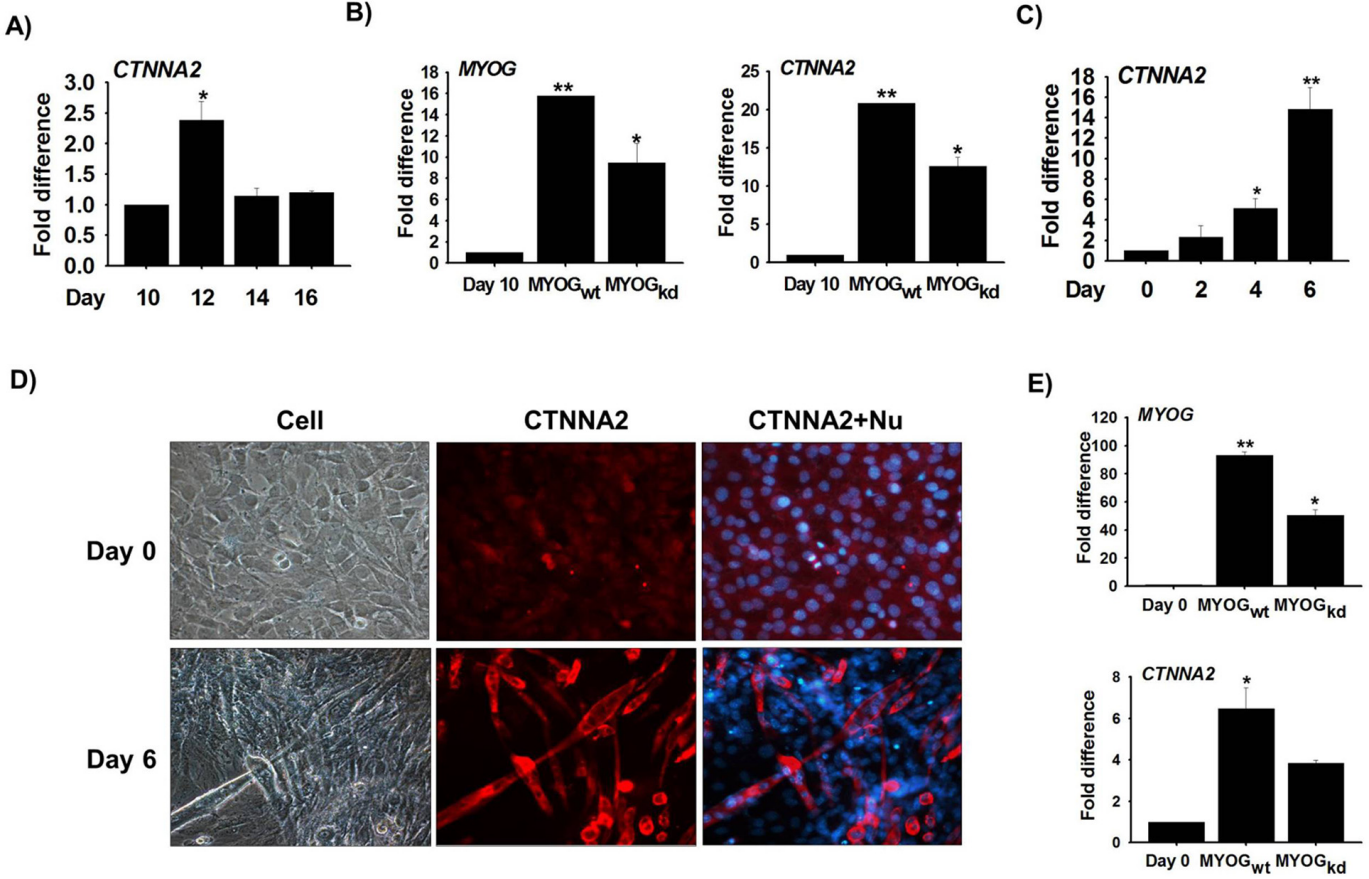
CTNNA2 expression during myogenesis in bovine MSCs and C2C12 cells. Bovine MSCs were cultured for 10, 12, 14, and 16 days in DMEM supplemented with 10% FBS and 1% penicillin/streptomycin (P/S). RNA was then isolated. **A)** CTNNA2 expression was analyzed by real-time RT-PCR and had increased on Day 12 compared to Day 10. **B)** MYOG gene expression was knocked down in bovine MSCs. MYOG and CTNNA2 expression was measured in MYOG knock-down cells. C2C12 cells were cultured with differentiation media for 0, 2, 4, and 6 days. mRNA expression was then analyzed by real-time RT-PCR. **C)** CTNNA2 expression was elevated on Day 6 compared to Day 0. **D)** Protein localization was observed by immunocytochemistry on Day 0 and 6. CTNNA2 protein was highly expressed inside myotubes on Day 6. **E)** MYOG expression was knocked down with shRNA in C2C12 cells that were cultured in differentiation media for 6 days. Gene expression was analyzed by real-time RT-PCR. Expression of MYOG and CTNNA2 was down-regulated in MYOG_kd_. The p-value indicated statistical significance of the data (p<0.05, mean ± standard deviation [S.D.], n = 3).

Based on our initial network analysis in which CTNNA2 was identified as the top hub gene with the maximum interacting partners (genes), an *in vitro* study was carried out to explore the functional significance of this gene in skeletal muscle development using primary bovine MSCs. After isolating the MSCs from bovine hind leg skeletal muscle, MSCs stained with Pax7 were used to determine cell purity. Around 85% of the total cell population was found to express Pax7. To elucidate the function of CTNNA2 during bovine MSC differentiation, changes in mRNA expression over time were evaluated. CTNNA2 expression was increased on Day 12 (Differentiation) compared to Day 10 (Proliferation) (**Fig 5A**). Correlation of CTNNA2 and MYOG expression was validated through RNA-Seq analysis (Lee et al., 2014). To establish the relationship between MYOG and CTNNA2, MYOG shRNA was transfected in MSCs. Expression of MYOG and CTNNA2 was decreased in MYOG_kd_ cells compared to MYOG_wt_ cells (**Fig 5B**). Results of these knock-down studies indicated that CTNNA2 expression is involved in myogenesis and is regulated by MYOG.

### CTNNA2 expression during differentiation and effects of MYOG, CTNNA2 knock-down in C2C12 cells

To confirm the data obtained from the bovine MSC studies, the expression of CTNNA2 in C2C12 cells was assessed. mRNA expression was measured in cells cultured with 2% FBS for 0, 2, 4, and 6 days. It was found that mRNA levels had gradually increased by day 6 compared to day 0 (**Fig 5C**).

Protein localization studies using immunocytochemistry revealed that CTNNA2 expression was highly restricted to the cytoplasm. Expression had increased on day 6 compared to day 0 (**Fig 5D**). To elucidate the correlation between CTNNA2 and MYOG expression in C2C12 cells, MYOG knock-down was performed with shRNA. Results of the experiment revealed that expression of both MYOG and CTNNA2 was down-regulated in MYOG_kd_ cells compared to MYOG_wt_ cells. Data from the knock-down studies demonstrated that expression of CTNNA2 is regulated by MYOG in C2C12 cells.

### Myotube formation and ECM gene expression in C2C12 cells with knocked down CTNNA2 expression

**Fig 6:**
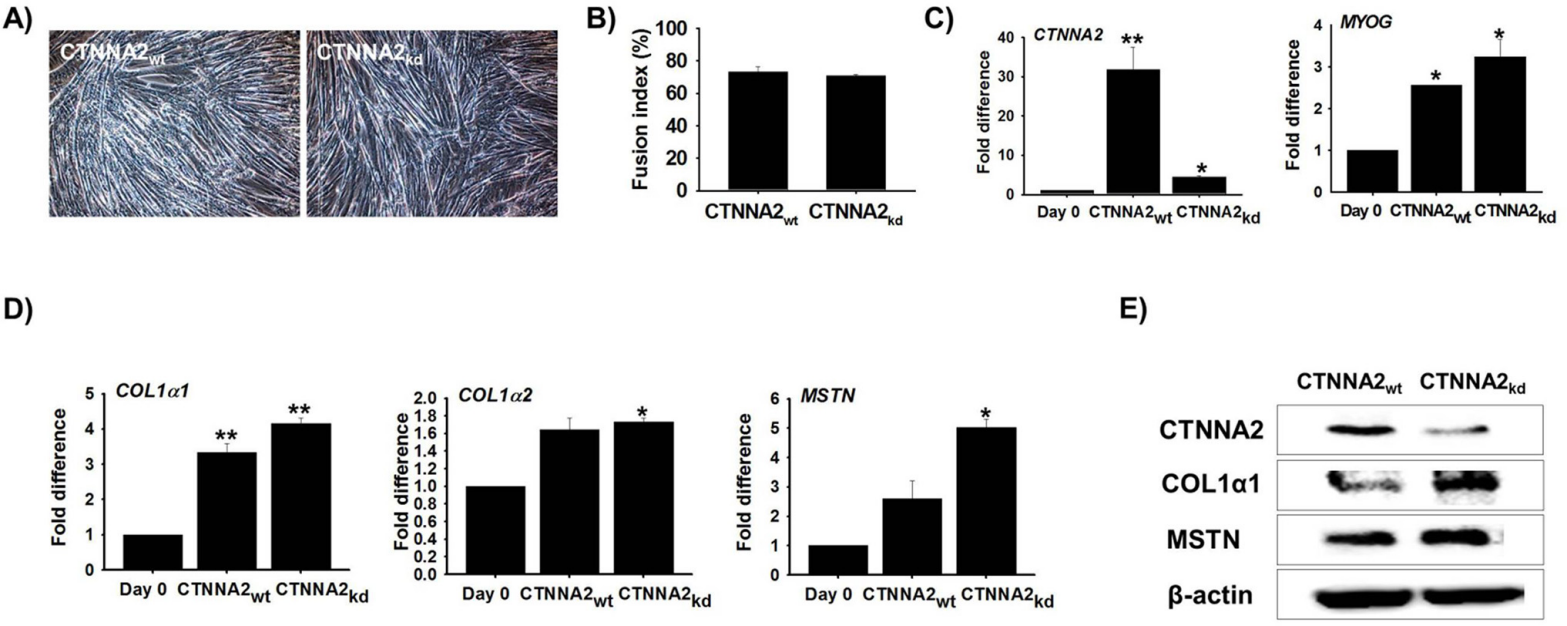
Myogenesis and associated genes evaluated by CTNNA2 knock-down. C2C12 cells were transfected with CTNNA2-specific siRNA and incubated with 2% FBS for 6 d. **A)** Morphology of the CTNNA2_wt_ and CTNNA2_kd_ cells was similar. **B)** The fusion index was determined and the values were similar for CTNNA2_wt_ and CTNNA2_kd_ cells. **C)** mRNA expression was analyzed by real-time RT-PCR. CTNNA2 expression was decreased in CTNNA2 knock-down cells. However, MYOG expression was similar in the CTNNA2_wt_ and CTNNA2_kd_ cells_._ **D)** Expression of ECM genes was analyzed by real-time RT-PCR. COL1α1, COL1α2, and MSTN gene expression was increased in CTNNA2_kd_ cells. In particular, MSTN expression was the highest among the four genes. **E)** Protein expression of CTNNA2, COL1α1, and MSTN in CTNNA2_kd_ was analyzed by Western blotting. CTNNA2 and ECM protein expression levels were similar to the mRNA expression patterns. The p-value indicates the statistical significance (p<0.05, mean ± S.D., n = 3).

C2C12 cells transfected with CTNNA2 siRNA (CTNNA2_kd_) were cultured with 2% FBS for 6 day. Cell morphology, tube formation, and fusion index values were similar for both cell (normal and knock-down) lines (**Fig 6A and 6B**)**.** CTNNA2 and MYOG mRNA expression was also analyzed by real-time RT-PCR. CTNNA2 expression was decreased substantially whereas MYOG expression was not significantly altered by knocking down CTNNA2 (CTNNA2_kd_; **Fig 6C**). Simultaneously, ECM is considered essential for myotube formation and the expression of muscle-specific gene products [41]. The expression of ECM genes (COL1α1, COL1α2, and MSTN) was increased during myogenesis (unpublished data). To establish the relationship between CTNNA2 and the ECM during myogenesis, mRNA and protein expression of COL1α1, COL1α2, and MSTN was measured in CTNNA2_kd_ cells. Both mRNA and protein expression of all three genes was significantly increased in CTNNA2_kd_ cells compared to CTNNA2_wt_ cells (**Fig 6D and 6E**). These results indicated that CTNNA2 helps maintain balance between the expression of MRF and ECM gene expression during muscle differentiation.

## Discussion

By definition, MSCs under the basal lamina are stem cells in muscle with the ability to self-renew and differentiate into myoblasts. Once satellite cells differentiate into myoblasts, they fuse into myofibers and contribute to myofiber growth. To obtain insight into the transcriptome of primary bovine MSCs, we recently performed an analysis with MYOG_kd_ samples using the RNA-Seq approach [23]. Generally, a typical high throughput analysis targets the identification of DEGs while a pathway analysis followed by experimental validation of a few selected genes (based on fold change in expression) is performed to measure functional enrichment. Knowing that DEGs may be equally or more significant for elucidating the process of skeletal muscle development, we created interaction networks of DEGs identified from MYOG_kd_ samples in order to obtain insight into the relationships with respect to one another. In the present study, we constructed three interaction networks (using up-regulated or down-regulated genes and a combination of all genes) from the list of DEGs for MYOG_kd_ primary bovine MSCs. As part of our previous study, we found that genes involved in cell division, DNA replication, and phosphate metabolic processes were differentially expressed [23]. The node degree of each gene in all three networks was calculated. It was observed that CTNNA2 had the highest node degree in ***Networks 1*** and ***3***, whereas CDC20 was the gene with highest node degree in ***Network 2***. Since CTNNA2 was found to have the highest node degree in two out of three networks, this gene was considered a hub gene in the present study. Therefore, its functional significance in skeletal muscle development was further explored.

The CTNNA2 gene encodes catenin alpha-2 or alpha N-catenin in humans and mice, but is recorded as an uncharacterized protein in the UniProt for bovine (UniProt IDs: P26232; Q61301). CTNNA2 protein acts as a linker between cadherin adhesion receptors and the cytoskeleton to control cell-cell adhesion and differentiation in the nervous system. This protein also regulates morphological plasticity of synapses as well as cerebellar and hippocampal lamination during development [42–44]. CTNNA2 is also part of a cell surface complex that includes Ig/FNIII proteins CDO and BOC, N- and M-cadherin (promyogenic cell adhesion molecules), and β- and α-catenin (cadherin-associated proteins) that may direct several aspects of myogenesis [45–48].

Recently, we identified CTNNA2 as a DEG that is down-regulated by at least 4-fold in MYOG_kd_ primary bovine MSCs using RNA-Seq analysis [23]. In **Fig 2,** it can be observed that CTNNA2 CTNNA2 (a cell adhesion protein) has diverse interacting partners including other cell adhesion molecules such as PLXNC1, CDK5R1, CDH18, COL15A1, and JAM2 (down-regulated) as well as IBSP, CLDN6, PCDHB11, and SELPLG (up-regulated). These cell adhesion molecules along with other genes as seen in **Fig 2**are involved in various biological processes such as collagen catabolism, skeletal muscle development, cell growth, and morphogenesis (**Table 3**). Moreover, we also observe cross-talk between these processes, thereby suggesting that one gene may be involved in various myogenic pathways (**Fig 2**). CTNNA2 expression was also increased during myogenesis. CTNNA2 and MYOG expression was analyzed in CTNNA2 and MYOG knock-down cells. CTNNA2 expression was decreased in MYOG_kd_. However, expression of MYOG was not significantly altered in CTNNA2 knock-down cells. Results of this experiment indicated that MYOG may be upstream of CTNNA2.

To further explore the role of CTNNA2 in myogenesis, we evaluated genes (MYOG, COL1α1, COL1α2, and MSTN) that are known to play substantial roles in skeletal muscle development [49–51]. A substantial increase in the expression of these genes (**Fig 6**) in CTNNA2_kd_ cells suggested that CTNNA2 holds a significant role in collagen catabolism, cell adhesion, and myogenesis. Collagen, proteoglycans, and adhesive glycoproteins are key components of the ECM, and are involved in ECM-receptor interactions as well as focal adhesion [50]. Explicit interactions among cells and ECM facilitated by transmembrane molecules or other cell surface-associated factors may directly or indirectly regulate cellular activities such as adhesion and migration [50].

Identifying the structure and function of biological networks is crucial for the exploration of biological phenomena. The interest in exploiting the use of network based studies to address key biological issues is ever increasing [52–54]. In this work, four functional modules or clusters were identified in ***Network 3*** using the GLay plugin for Cytoscape. Additionally, the functional enrichment of each module in the network was explored using the DAVID functional annotation tool. Our analysis demonstrated that among the 10 most significant enrichments, cluster 1 homeostasis was highly overrepresented. Some of the genes grouped under homeostasis by the DAVID functional analysis encode channel proteins such as ryanodine receptor 1 (RYR1) and sodium channel protein type 1 subunit alpha (SCN1A). RYR1 is an indispensable factor for maintaining calcium homeostasis in mammalian skeletal muscle. Inactivation of this protein is lethal at birth in mice and mutations of the RYR1 gene in humans are associated various muscle disorders [51]. Similarly, voltage-gated sodium channels are involved in the early increase and consequent transmission of action potential in skeletal muscle [55]. Cluster 2 was enriched in processes related to the cell cycle and DNA replication. It included genes that encode various cell division homologue proteins such as cell division cycle 45 (CDC45), CDC20, and CDC6. The third cluster in the network was enriched in phosphorous metabolic processes. The community structure of our network concurs with results of our recent work on MYOG_kd_ primary bovine MSCs [23].

Molecular target discovery and targeted therapeutics have become indispensable ever since the advancement of bioinformatics [56]. Although the present study highlights the importance of *in silico* approaches for assigning novel role to genes such as CTNNA2 in myogenesis, at the same time our findings open up several avenues that emphasize the importance of predicting how genes interact with each other to regulate homeostasis. It is also apparent that *in silico*-predicted results are highly correlated with experimental data. Altogether, understanding the structure and function of DEGs is essential not only for elucidating their roles during proliferation and differentiation, but can also be useful for predicting the binding specificities of genes to counteract several muscular dystrophies by designing potent inhibitors against target genes.

## Supporting Information

S1 Table A) shRNA information B) siRNA information. (DOCX)

S2 Table Primer information. (DOCX)

S3 Table Functional enrichment of down-regulated and 50 related genes in the network as reported by GeneMANIA. (DOCX)

S4 Table Functional enrichment of up-regulated and 50 related genes in the network as reported by GeneMANIA. (DOCX)

S5 Table 136 enriched GO terms in cluster 1 as detected by Glay. (DOCX)

S6 Table 93 enriched GO terms in cluster 2 as detected by GLay. (DOCX)

S7 Table A) Enriched GO terms in cluster 1 detected by MCODE, B) Enriched GO terms in clusters detected by MCL. (DOCX)

## References

[1] Meadows E, Cho JH, Flynn JM, Klein WH (2008) Myogenin regulates a distinct genetic program in adult muscle stem cells. Dev Biol 322: 406–14. 10.1016/j.ydbio.2008.07.024 18721801

[2] Olson EN, Brennan TJ, Chakraborty T, Cheng TC, Cserjesi P, Edmondson D, et al (1991) Molecular control of myogenesis: antagonism between growth and differentiation. Mol Cell Biochem 104: 7–13. 1922004 10.1007/BF00229797

[3] Mohun T. Muscle differentiation. Curr Opin Cell Biol 4: 923–928. 1485959 10.1016/0955-0674(92)90119-w

[4] Andrés V, Walsh K (1996) Myogenin expression, cell cycle withdrawal, and phenotypic differentiation are temporally separable events that precede cell fusion upon myogenesis. J Cell Biol 132: 657–66. 8647896 10.1083/jcb.132.4.657 PMC2199863

[5] Moran JL, Li Y, Hill AA, Mounts WM, Miller CP (2002) Gene expression changes during mouse skeletal myoblast differentiation revealed by transcriptional profiling. Physiol Genomics 10: 103–11. 12181367 10.1152/physiolgenomics.00011.2002

[6] Janot M, Audfray A, Loriol C, Germot A, Maftah A, Dupuy F (2009) Glycogenome expression dynamics during mouse C2C12 myoblast differentiation suggests a sequential reorganization of membrane glycoconjugates. BMC Genomics 10: 483 10.1186/1471-2164-10-483 19843320 PMC2772862

[7] Rajan S, Chu Pham Dang H, Djambazian H, Zuzan H, Ketela T, et al (2012) Analysis of early C2C12 myogenesis identifies stably and differentially expressed transcriptional regulators whose knock-down inhibits myoblast differentiation. Physiol Genomics 2: 183–197. 10.1152/physiolgenomics.00093.2011 22147266

[8] Rudnicki MA, Schnegelsberg PNJ, Stead RH, Braun T, Arnold HH, Jaenisch R (1993) MyoD or Myf-5 is required for the formation of skeletal muscle. Cell 75: 1351–1359. 8269513 10.1016/0092-8674(93)90621-v

[9] Rawls A, Morris JH, Rudnicki M, Braun T, Arnold HH, Klein WH, et al (1995) Myogenin's functions do not overlap with those of MyoD or Myf-5 during mouse embryogenesis. Dev Biol 172: 37–50. 7589813 10.1006/dbio.1995.0004

[10] Cao Y, Kumar RM, Penn BH, Berkes CA, Kooperberg C, Boyer LA, et al (2006) Global and gene-specific analyses show distinct roles for Myod and Myog at a common set of promoters. EMBO J 25: 502–511. 16437161 10.1038/sj.emboj.7600958 PMC1383539

[11] Wang Y, Jaenisch R (1997) Myogenin can substitute for Myf5 in promoting myogenesis but less efficiently. Development 124: 2507–2513. 9216993 10.1242/dev.124.13.2507

[12] Rajan S, Chu Pham Dang H, Djambazian H, Zuzan H, Fedyshyn Y, Ketela T, et al (2012) Analysis of early C2C12 myogenesis identifies stably and differentially expressed transcriptional regulators whose knock-down inhibits myoblast differentiation. Physiol Genomics 2: 183–197. 10.1152/physiolgenomics.00093.2011 22147266

[13] Kablar B, Asakura A, Krastel K, Ying C, May LL, Goldhamer DJ, et al (1998) MyoD and Myf-5 define the specification of musculature of distinct embryonic origin. Biochem Cell Biol 76: 1079–1091. 10392718

[14] Kassar-Duchossoy L, Gayraud-Morel B, Gomès D, Rocancourt D, Buckingham M, Shinin V, et al (2004) Mrf4 determines skeletal muscle identity in Myf5:Myod double-mutant mice. Nature 431: 466–471. 15386014 10.1038/nature02876

[15] Bischoff R. (1994) The satellite cell and muscle regeneration Engel A. (Ed.), Myology, Mc-Graw Hill, New York 1: 97–118.

[16] Charge SB, Rudnicki MA (2004) Cellular and molecular regulation of muscle regeneration. Physiol Rev 84: 209–238. 14715915 10.1152/physrev.00019.2003

[17] Cossu G, Molinaro M (1987) Cell heterogeneity in the myogenic lineage. Curr Top Dev Biol 23: 185–208. 3330504 10.1016/s0070-2153(08)60625-0

[18] Gibson MC, Schultz E (1983) Age-related differences in absolute numbers of skeletal muscle satellite cells. Muscle Nerve 6: 574–580. 6646160 10.1002/mus.880060807

[19] Schultz E, Jaryszak DL, Valliere CR (1985) Response of satellite cells to focal skeletal muscle injury. Muscle Nerve 8: 217–222. 4058466 10.1002/mus.880080307

[20] Lee EJ, Lee HJ, Kamli MR, Pokharel S, Bhat AR, Lee YH, et al (2012) Depot-specific gene expression profiles during differentiation and transdifferentiation of bovine muscle satellite cells, and differentiation of preadipocytes. Genomics 100: 195–202. 10.1016/j.ygeno.2012.06.005 22728265

[21] Lee EJ, Kamli MR, Pokharel S, Malik A, Tareq KM, Roof BA, et al (2013) Expressed sequence tags for bovine muscle satellite cells, myotube formed-cells and adipocyte-like cells. PLOS ONE 8: e79780 10.1371/journal.pone.0079780 24224006 PMC3818215

[22] Lee EJ, Bhat AR, Kaml MR, Pokharel S, Chun T, Lee YH, et al (2013) Transthyretin is a key regulator of myoblast differentiation. PLOS ONE 8: e63627 10.1371/journal.pone.0063627 23717457 PMC3661549

[23] Lee EJ, Malik A, Pokharel S, Ahmad S, Mir BA, Cho KH, et al (2014) Identification of genes differentially expressed in myogenin knock-down bovine muscle satellite cells during differentiation through RNA sequencing analysis. PLOS ONE 9: e92447 doi: 10.1371/journal.pone. 0092447 24647404 10.1371/journal.pone.0092447 PMC3960249

[24] Sterrenburg E, Turk R, 't Hoen PA, van Deutekom JC, Boer JM, van Ommen GJ, et al (2004) Large-scale gene expression analysis of human skeletal myoblast differentiation. Neuromuscul Disord 14: 507–518. 15336692 10.1016/j.nmd.2004.03.008

[25] Shen X, Collier JM, Hlaing M, Zhang L, Delshad EH, Bristow J, et al (2003) Genome-wide examination of myoblast cell cycle withdrawal during differentiation. Dev Dyn 1: 128–138. 12508234 10.1002/dvdy.10200

[26] Tomczak KK, Marinescu VD, Ramoni MF, Sanoudou D, Montanaro F, Han M, et al (2004) Expression profiling and identification of novel genes involved in myogenic differentiation. FASEB J 18: 403–405. 14688207 10.1096/fj.03-0568fje

[27] Liu QC, Zha XH, Faralli H, Yin H, Louis-Jeune C, Perdiguero E, et al (2012) Comparative expression profiling identifies differential roles for Myogenin and p38α MAPK signaling in myogenesis. J Mol Cell Biol 4: 386–397. 10.1093/jmcb/mjs045 22847234 PMC4580549

[28] Wicik Z, Sadkowski T, Jank M, Motyl T (2011) The transcriptomic signature of myostatin inhibitory influence on the differentiation of mouse C2C12 myoblasts. Pol J Vet Sci 4: 643–652. 22439337 10.2478/v10181-011-0095-7

[29] Langmead B, Trapnell C, Pop M, Salzberg SL (2010) Ultrafast and memory-efficient alignment of short DNA sequences to the human genome. Genome Biol 10: R25 10.1186/gb-2009-10-3-r25 19261174 PMC2690996

[30] Warde-Farley D, Donaldson SL, Comes O, Zuberi K, Badrawi R, Chao P, et al (2010) The GeneMANIA prediction server: biological network integration for gene prioritization and predicting gene function. Nucleic Acids Res 38: W214–20. 10.1093/nar/gkq537 20576703 PMC2896186

[31] Albert R (2005) Scale-free networks in cell biology. J Cell Sci 118: 4947–4957. 16254242 10.1242/jcs.02714

[32] Wu XR, Zhu Y, Li Y (2005) Analyzing protein interaction networks via random graph model. Int. J. Inf. Technol 11: 125–132.

[33] Smoot ME, Ono K, Ruscheinski J, Wang PL, Ideker T (2001) Cytoscape 2.8: new features for data integration and network visualization. Bioinformatics 27: 431–432. 10.1093/bioinformatics/btq675 21149340 PMC3031041

[34] van Dongen S. Graph Clustering by Flow Simulation. Unpublished doctoral dissertation. Centre for Mathematics and Computer Science, University of Utrecht, The Netherlands. 2000a

[35] van Dongen S. MCL—an algorithm for clustering graphs. Available: http://micans.org/mcl/ 2000b.

[36] Bader GD, Hogue CW (2003) An automated method for finding molecular complexes in large protein interaction networks. BMC Bioinformatics 4: 2 12525261 10.1186/1471-2105-4-2 PMC149346

[37] Newman MEJ, Girvan M (2004) Finding and evaluating community structure in networks. Phys Rev E 69: 026113 14995526 10.1103/PhysRevE.69.026113

[38] Morris JH, Apeltsin L, Newman AM, Baumbach J, Wittkop T, Su G, et al (2011) clusterMaker: a multi-algorithm clustering plugin for Cytoscape. BMC Bioinformatics 12: 436 10.1186/1471-2105-12-436 22070249 PMC3262844

[39] Su G, Kuchinsky A, Morris JH, States DJ, Meng F (2010) GLay: community structure analysis of biological networks. Bioinformatics 26: 3135–3137. 10.1093/bioinformatics/btq596 21123224 PMC2995124

[40] Lee SH, Park BH, Sharma A, Dang CG, Lee SS Choi TJ et al (2014) Hanwoo cattle: origin, domestication, breeding strategies and genomic selection. Journal of Animal Science and Technology 56: 2 10.1186/2055-0391-56-2 26290691 PMC4534185

[41] Dinh P, Sotiriou C, Piccart MJ (2007) The evolution of treatment strategies: aiming at the target. Breast 16: S10–6. 17764940 10.1016/j.breast.2007.07.032

[42] Park C, Falls W, Finger JH, Longo-Guess CM, Ackerman SL (2002) Deletion in Catna2, encoding alpha N-catenin, causes cerebellar and hippocampal lamination defects and impaired startle modulation. Nat Genet 31: 279–84. 12089526 10.1038/ng908

[43] Togashi H, Abe K, Mizoguchi A, Takaoka K, Chisaka O, Takeichi M (2002) Cadherin regulates dendritic spine morphogenesis. Neuron 35: 77–89. 12123610 10.1016/s0896-6273(02)00748-1

[44] Abe T, Takagi N, Nakano M, Furuya M, Takeo S (2004) Altered Bad localization and interaction between Bad and Bcl-xL in the hippocampus after transient global ischemia. Brain Res 1009: 159–68. 15120593 10.1016/j.brainres.2004.03.003

[45] Kang JS, Mulieri PJ, Hu Y, Taliana L, Krauss RS (2002) BOC, an Ig superfamily member, associates with CDO to positively regulate myogenic differentiation. EMBO J 21: 114–24. 11782431 10.1093/emboj/21.1.114 PMC125805

[46] Kang JS, Feinleib JL, Knox S, Ketteringham MA, Krauss RS (2003) Promyogenic members of the Ig and cadherin families associate to positively regulate differentiation. Proc Natl Acad Sci U S A. 100: 3989–94. 12634428 10.1073/pnas.0736565100 PMC153035

[47] Kang JS, Yi MJ, Zhang W, Feinleib JL, Cole F, Krauss RS (2004) Netrins and neogenin promote myotube formation. J Cell Biol 167: 493–504. 15520228 10.1083/jcb.200405039 PMC2172498

[48] Reactome pathway: http://www.reactome.org/PathwayBrowser/#REACT_21402

[49] Andrés V, Walsh K (1996) Myogenin expression, cell cycle withdrawal, and phenotypic differentiation are temporally separable events that precede cell fusion upon myogenesis. J Cell Biol 4: 657–66. 8647896 10.1083/jcb.132.4.657 PMC2199863

[50] Li Y, Xu Z, Li H, Xiong Y, Zuo B (2012) Differential transcriptional analysis between red and white skeletal muscle of Chinese Meishan pigs. Int J Biol Sci 6: 350–360. 20617128 10.7150/ijbs.6.350 PMC2899453

[51] Monnier N, Ferreiro A, Marty I, Labarre-Vila A, Mezin P, Lunardi J (2003) A homozygous splicing mutation causing a depletion of skeletal muscle RYR1 is associated with multi-minicore disease congenital myopathy with ophthalmoplegia. Hum Mol Genet 12: 1171–8. 12719381 10.1093/hmg/ddg121

[52] Lee J, Lee J (2013) Hidden information revealed by optimal community structure from a protein-complex bipartite network improves protein function prediction. PLOS ONE 8:e60372 doi: 10.1371/journal.pone.0060372. 23577106 23577106 10.1371/journal.pone.0060372 PMC3618231

[53] Malik A, Lee J, Lee J (2014) Community-based network study of protein-carbohydrate interactions in plant lectins using glycan array data. PLOS ONE 9:e95480 10.1371/journal.pone.0095480 24755681 PMC3995809

[54] Firoz A, Malik A, Singh SK, Jha V, Ali A (2014) Comparative Analysis of Glycogene Expression in Different Mouse Tissues Using RNA-Seq Data. Int J Genomics 837365. doi: 10.1155/2014/837365. 25121089 10.1155/2014/837365 PMC4121153 25121089

[55] Desaphy JF, De Luca A, Camerino DC (1998) Blockade by cAMP of native sodium channels of adult rat skeletal muscle fibers. Am J Physiol 275: C1465–72. 9843707 10.1152/ajpcell.1998.275.6.C1465

[56] Dinh P, Sotiriou C, Piccart MJ (2007) The evolution of treatment strategies: aiming at the target. Breast. 16: S10–6. 17764940 10.1016/j.breast.2007.07.032

