# Supplementary material for: Network Analysis for the Identification of Differentially Expressed Hub Genes Using Myogenin Knock-down Muscle Satellite Cells
Source: PLoS One. 2015 Jul 22;10(7):e0133597. doi: 10.1371/journal.pone.0133597 (PMC4511796; doi:10.1371/journal.pone.0133597)
Supplement: S1 Table — (DOCX) [file pone.0133597.s001.docx]

**S1 Table.**

**A) shRNA information**

| **shRNA** |  | **Sequence** | |
| --- | --- | --- | --- |
| **MYOG** | MYOG shRNA Plasmid (m) is a pool of 3 different shRNA plasmids | | |
|  | sc-35992-SHA | Hairpin sequence | GATCCGCATGCAAGGTGTGTAAGATTCAAGAGATCTTACACACCTTGCATGCTTTTT |
|  |  | Sense | GCAUGCAAGGUGUGUAAGAtt |
|  |  | Antisense | UCUUACACACCUUGCAUGCtt |
|  | sc-35992-SHB | Hairpin sequence | GATCCCTGTCCTGATGTCCAGAAATTCAAGAGATTTCTGGACATCAGGACAGTTTTT |
|  |  | Sense | CUGUCCUGAUGUCCAGAAAtt |
|  |  | Antisense | UUUCUGGACAUCAGGACAGtt |
|  | sc-35992-SHC | Hairpin sequence | GATCCCCCATTCACATAAGGCTAATTCAAGAGATTAGCCTTATGTGAATGGGTTTTT |
|  |  | Sense | CCCAUUCACAUAAGGCUAAtt |
|  |  | Antisense | UUAGCCUUAUGUGAAUGGGtt |

**B) siRNA information**

| Gene | siRNA | Target sequence |
| --- | --- | --- |
| CTNNA2si | ON-TARGETplus SMARTpool siRNA J-048969-09, Ctnna2 | GCUCAGUGGUGGAACGUUA |
|  | ON-TARGETplus SMARTpool siRNA J-048969-10, Ctnna2 | GAGGAGACAGCUUCGGAAA |
|  | ON-TARGETplus SMARTpool siRNA J-048969-11, Ctnna2 | CUACAAGAGCCAACCGAGA |
|  | ON-TARGETplus SMARTpool siRNA J-048969-12, Ctnna2 | GGUGAAAGCGUCUUAUGUA |
