# Supplementary material for: Network Analysis for the Identification of Differentially Expressed Hub Genes Using Myogenin Knock-down Muscle Satellite Cells
Source: PLoS One. 2015 Jul 22;10(7):e0133597. doi: 10.1371/journal.pone.0133597 (PMC4511796; doi:10.1371/journal.pone.0133597)
Supplement: S2 Table — (DOCX) [file pone.0133597.s002.docx]

**S2 Table. Primer information**

| **Species** | **Gene** | **Product size (bp)** | **Tm (°C )** | **Sequence (F)** | **Sequence (R)** |
| --- | --- | --- | --- | --- | --- |
| Bovine | GAPDH | 211 | 52 | 5'-gggtcatctctgcacct-3' | 5'-acagtcttctgggtggcagt-3' |
|  | MYOG | 197 | 57 | 5'-tgggcgtgtaaggtgtgtaa-3' | 5'-tgcaggcgctctatgtactg-3' |
|  | CTNNA2 | 224 | 56 | 5'-aaggtgagaccatgcgaatc-3' | 5'-tgcaaggtcttgctcatttg-3' |
| Mouse | GAPDH | 155 | 55 | 5'-tgctggtgctgagtatgtcg-3' | 5'-caagcagttggtggtacagg-3' |
|  | MYOG | 185 | 185 | 5'-tccagtacattgagcgccta-3' | 5'-caaatgatctcctgggttgg-3' |
|  | CTNNA2 | 195 | 55 | 5’-Cctctcaacattgcgattga-3’ | 5’-ggcatgttcacggaaaactt-3' |
|  | COL1α1 | 244 | 59 | 5'-ctttgcttcccagatgtcct-3' | 5'-ccccatcatctccattcttg-3' |
|  | COL1α2 | 247 | 59 | 5'-ctgatggcagagctggtgta-3' | 5'-atgttgccagcttcacctct-3' |
|  | MSTN | 163 | 59 | 5'-acgctaccacggaaacaatc-3' | 5'-ggagtcttgacgggtctgag-3' |
