# Supplementary material for: Network Analysis for the Identification of Differentially Expressed Hub Genes Using Myogenin Knock-down Muscle Satellite Cells
Source: PLoS One. 2015 Jul 22;10(7):e0133597. doi: 10.1371/journal.pone.0133597 (PMC4511796; doi:10.1371/journal.pone.0133597)
Supplement: S3 Table — (DOCX) [file pone.0133597.s003.docx]

**S3 Table.** Functional enrichment of down-regulated and 50 related genes in the network as reported by GeneMANIA

| Feature | FDR |
| --- | --- |
| transforming growth factor beta receptor signaling pathway | 0.07319826 |
| response to transforming growth factor beta stimulus | 0.08196842 |
| cellular response to transforming growth factor beta stimulus | 0.08196842 |
| enzyme inhibitor activity | 0.4370629 |
| transmembrane receptor protein serine/threonine kinase signaling pathway | 0.4370629 |
| kinase activator activity | 0.4370629 |
| regulation of cellular response to growth factor stimulus | 0.4370629 |
| heart morphogenesis | 0.4688191 |
| viral genome expression | 0.4688191 |
| viral transcription | 0.4688191 |
