# Supplementary material for: Network Analysis for the Identification of Differentially Expressed Hub Genes Using Myogenin Knock-down Muscle Satellite Cells
Source: PLoS One. 2015 Jul 22;10(7):e0133597. doi: 10.1371/journal.pone.0133597 (PMC4511796; doi:10.1371/journal.pone.0133597)
Supplement: S4 Table — (DOCX) [file pone.0133597.s004.docx]

**S4 Table.** Functional enrichment of up-regulated and 50 related genes in the network as reported by GeneMANIA

| Function | FDR |
| --- | --- |
| Regulation of cell cycle process | 7.53E-09 |
| cell cycle checkpoint | 7.95E-09 |
| interphase of mitotic cell cycle | 7.95E-09 |
| interphase | 8.91E-09 |
| DNA replication | 2.16E-08 |
| response to zinc ion | 2.16E-08 |
| mitosis | 0.000000114 |
| nuclear division | 0.000000114 |
| regulation of mitotic cell cycle | 0.000000136 |
| M phase of mitotic cell cycle | 0.000000215 |
| 12 organelle fission | 0.000000353 |
| 13 cellular response to metal ion | 0.000000516 |
| 14 S phase of mitotic cell cycle | 0.000000571 |
| 15 cellular response to inorganic substance | 0.00000071 |
| 16 DNA strand elongation involved in DNA replication | 0.000000738 |
| 17 S phase | 0.000000965 |
| 18 DNA strand elongation | 0.00000149 |
| 19 response to cadmium ion | 0.00000292 |
