# Supplementary material for: Network Analysis for the Identification of Differentially Expressed Hub Genes Using Myogenin Knock-down Muscle Satellite Cells
Source: PLoS One. 2015 Jul 22;10(7):e0133597. doi: 10.1371/journal.pone.0133597 (PMC4511796; doi:10.1371/journal.pone.0133597)
Supplement: S5 Table — (DOCX) [file pone.0133597.s005.docx]

**S5 Table. 136** **enriched GO terms in cluster 1 as detected by Glay**

| Term | PValue |
| --- | --- |
| GO:0050900~leukocyte migration | 1.31604E-05 |
| GO:0009611~response to wounding | 1.5127E-05 |
| GO:0055080~cation homeostasis | 4.64822E-05 |
| GO:0050801~ion homeostasis | 5.68711E-05 |
| GO:0006873~cellular ion homeostasis | 6.74522E-05 |
| GO:0055082~cellular chemical homeostasis | 8.13357E-05 |
| GO:0006928~cell motion | 0.000111972 |
| GO:0016477~cell migration | 0.000128237 |
| GO:0055066~di-, tri-valent inorganic cation homeostasis | 0.000128787 |
| GO:0030003~cellular cation homeostasis | 0.000227307 |
| GO:0008015~blood circulation | 0.000273314 |
| GO:0003013~circulatory system process | 0.000273314 |
| GO:0048666~neuron development | 0.000278783 |
| GO:0007155~cell adhesion | 0.000289306 |
| GO:0022610~biological adhesion | 0.00029508 |
| GO:0030005~cellular di-, tri-valent inorganic cation homeostasis | 0.000333985 |
| GO:0048545~response to steroid hormone stimulus | 0.000352636 |
| GO:0048870~cell motility | 0.000362655 |
| GO:0051674~localization of cell | 0.000362655 |
| GO:0048878~chemical homeostasis | 0.000795784 |
| GO:0019725~cellular homeostasis | 0.00079713 |
| GO:0006874~cellular calcium ion homeostasis | 0.00104397 |
| GO:0055074~calcium ion homeostasis | 0.001261275 |
| GO:0043066~negative regulation of apoptosis | 0.001359754 |
| GO:0007179~transforming growth factor beta receptor signaling pathway | 0.001445824 |
| GO:0043069~negative regulation of programmed cell death | 0.001533732 |
| GO:0016049~cell growth | 0.001557434 |
| GO:0060548~negative regulation of cell death | 0.001584143 |
| GO:0009719~response to endogenous stimulus | 0.00158549 |
| GO:0030595~leukocyte chemotaxis | 0.001667131 |
| GO:0030595~leukocyte chemotaxis | 0.001667131 |
| GO:0030595~leukocyte chemotaxis | 0.001667131 |
| GO:0006875~cellular metal ion homeostasis | 0.001680443 |
| GO:0042981~regulation of apoptosis | 0.0018016 |
| GO:0009725~response to hormone stimulus | 0.001871507 |
| GO:0006954~inflammatory response | 0.001975165 |
| GO:0060326~cell chemotaxis | 0.002031882 |
| GO:0060326~cell chemotaxis | 0.002031882 |
| GO:0060326~cell chemotaxis | 0.002031882 |
| GO:0043067~regulation of programmed cell death | 0.002038545 |
| GO:0010941~regulation of cell death | 0.002134009 |
| GO:0000904~cell morphogenesis involved in differentiation | 0.002172916 |
| GO:0055065~metal ion homeostasis | 0.002283195 |
| GO:0008361~regulation of cell size | 0.002385499 |
| GO:0030574~collagen catabolic process | 0.002529725 |
| GO:0001666~response to hypoxia | 0.002667928 |
| GO:0031175~neuron projection development | 0.003104308 |
| GO:0030182~neuron differentiation | 0.003245421 |
| GO:0070482~response to oxygen levels | 0.003542957 |
| GO:0042592~homeostatic process | 0.003953136 |
| GO:0040007~growth | 0.004068729 |
| GO:0007626~locomotory behavior | 0.005028103 |
| GO:0042060~wound healing | 0.005233339 |
| GO:0044243~multicellular organismal catabolic process | 0.00542767 |
| GO:0007610~behavior | 0.005904142 |
| GO:0002684~positive regulation of immune system process | 0.00608683 |
| GO:0002684~positive regulation of immune system process | 0.00608683 |
| GO:0002684~positive regulation of immune system process | 0.00608683 |
| GO:0032963~collagen metabolic process | 0.006701396 |
| GO:0002237~response to molecule of bacterial origin | 0.006905433 |
| GO:0006935~chemotaxis | 0.007034093 |
| GO:0042330~taxis | 0.007034093 |
| GO:0001568~blood vessel development | 0.007283591 |
| GO:0001944~vasculature development | 0.008494628 |
| GO:0044259~multicellular organismal macromolecule metabolic process | 0.008919609 |
| GO:0048514~blood vessel morphogenesis | 0.009364821 |
| GO:0000902~cell morphogenesis | 0.011081042 |
| GO:0010243~response to organic nitrogen | 0.011375259 |
| GO:0014823~response to activity | 0.011569402 |
| GO:0014823~response to activity | 0.011569402 |
| GO:0002526~acute inflammatory response | 0.011794109 |
| GO:0002526~acute inflammatory response | 0.011794109 |
| GO:0040017~positive regulation of locomotion | 0.011794109 |
| GO:0040017~positive regulation of locomotion | 0.011794109 |
| GO:0002526~acute inflammatory response | 0.011794109 |
| GO:0033273~response to vitamin | 0.013330313 |
| GO:0048820~hair follicle maturation | 0.013548977 |
| GO:0032535~regulation of cellular component size | 0.013549274 |
| GO:0050878~regulation of body fluid levels | 0.013911608 |
| GO:0007178~transmembrane receptor protein serine/threonine kinase signaling pathway | 0.014388944 |
| GO:0044236~multicellular organismal metabolic process | 0.014511406 |
| GO:0051971~positive regulation of transmission of nerve impulse | 0.014511406 |
| GO:0016337~cell-cell adhesion | 0.015101157 |
| GO:0043627~response to estrogen stimulus | 0.015526183 |
| GO:0031646~positive regulation of neurological system process | 0.016727694 |
| GO:0014075~response to amine stimulus | 0.016727694 |
| GO:0010647~positive regulation of cell communication | 0.016754833 |
| GO:0001525~angiogenesis | 0.017286104 |
| GO:0001525~angiogenesis | 0.017286104 |
| GO:0007599~hemostasis | 0.017342232 |
| GO:0002687~positive regulation of leukocyte migration | 0.017910372 |
| GO:0031667~response to nutrient levels | 0.020237159 |
| GO:0006090~pyruvate metabolic process | 0.020388071 |
| GO:0051240~positive regulation of multicellular organismal process | 0.020549368 |
| GO:0051240~positive regulation of multicellular organismal process | 0.020549368 |
| GO:0032496~response to lipopolysaccharide | 0.022270117 |
| GO:0043200~response to amino acid stimulus | 0.022781157 |
| GO:0032989~cellular component morphogenesis | 0.022984144 |
| GO:0010033~response to organic substance | 0.023149529 |
| GO:0051384~response to glucocorticoid stimulus | 0.023226662 |
| GO:0050863~regulation of T cell activation | 0.023618225 |
| GO:0006959~humoral immune response | 0.024207859 |
| GO:0006952~defense response | 0.024629893 |
| GO:0030593~neutrophil chemotaxis | 0.025397789 |
| GO:0006916~anti-apoptosis | 0.025427242 |
| GO:0014070~response to organic cyclic substance | 0.026823028 |
| GO:0002683~negative regulation of immune system process | 0.028381539 |
| GO:0002683~negative regulation of immune system process | 0.028381539 |
| GO:0044057~regulation of system process | 0.028790501 |
| GO:0051346~negative regulation of hydrolase activity | 0.028924039 |
| GO:0048812~neuron projection morphogenesis | 0.029345715 |
| GO:0031960~response to corticosteroid stimulus | 0.030619119 |
| GO:0002685~regulation of leukocyte migration | 0.030974694 |
| GO:0042493~response to drug | 0.031351101 |
| GO:0002443~leukocyte mediated immunity | 0.031775884 |
| GO:0030030~cell projection organization | 0.033017214 |
| GO:0009991~response to extracellular stimulus | 0.034155702 |
| GO:0030335~positive regulation of cell migration | 0.035398816 |
| GO:0045861~negative regulation of proteolysis | 0.036985601 |
| GO:0030162~regulation of proteolysis | 0.037267634 |
| GO:0006811~ion transport | 0.038914841 |
| GO:0019228~regulation of action potential in neuron | 0.039068719 |
| GO:0002252~immune effector process | 0.039096648 |
| GO:0030308~negative regulation of cell growth | 0.039251594 |
| GO:0010332~response to gamma radiation | 0.040145028 |
| GO:0030155~regulation of cell adhesion | 0.042343804 |
| GO:0009612~response to mechanical stimulus | 0.042801399 |
| GO:0001101~response to acid | 0.043402574 |
| GO:0048738~cardiac muscle tissue development | 0.044732442 |
| GO:0007584~response to nutrient | 0.045749311 |
| GO:0050777~negative regulation of immune response | 0.046754932 |
| GO:0006094~gluconeogenesis | 0.046754932 |
| GO:0048584~positive regulation of response to stimulus | 0.047047098 |
| GO:0048584~positive regulation of response to stimulus | 0.047047098 |
| GO:0051272~positive regulation of cell motion | 0.047648424 |
| GO:0045792~negative regulation of cell size | 0.049137396 |
