# Supplementary material for: Network Analysis for the Identification of Differentially Expressed Hub Genes Using Myogenin Knock-down Muscle Satellite Cells
Source: PLoS One. 2015 Jul 22;10(7):e0133597. doi: 10.1371/journal.pone.0133597 (PMC4511796; doi:10.1371/journal.pone.0133597)
Supplement: S6 Table — (DOCX) [file pone.0133597.s006.docx]

**S6 Table. 93 enriched GO terms in cluster 2 as detected by GLay**

| Term | PValue |
| --- | --- |
| GO:0007049~cell cycle | 2.61097E-15 |
| GO:0000278~mitotic cell cycle | 1.61351E-13 |
| GO:0022403~cell cycle phase | 2.33412E-12 |
| GO:0022402~cell cycle process | 4.30703E-12 |
| GO:0007067~mitosis | 4.61724E-11 |
| GO:0000280~nuclear division | 4.61724E-11 |
| GO:0000087~M phase of mitotic cell cycle | 6.31756E-11 |
| GO:0048285~organelle fission | 9.25736E-11 |
| GO:0000279~M phase | 1.8784E-10 |
| GO:0006260~DNA replication | 2.45326E-09 |
| GO:0051301~cell division | 6.59754E-09 |
| GO:0006259~DNA metabolic process | 2.54255E-08 |
| GO:0006259~DNA metabolic process | 2.54255E-08 |
| GO:0006270~DNA replication initiation | 2.78986E-08 |
| GO:0006261~DNA-dependent DNA replication | 6.47303E-07 |
| GO:0006268~DNA unwinding during replication | 2.2869E-05 |
| GO:0051439~regulation of ubiquitin-protein ligase activity during mitotic cell cycle | 3.18814E-05 |
| GO:0043161~proteasomal ubiquitin-dependent protein catabolic process | 4.60892E-05 |
| GO:0010498~proteasomal protein catabolic process | 4.60892E-05 |
| GO:0051438~regulation of ubiquitin-protein ligase activity | 5.87845E-05 |
| GO:0006511~ubiquitin-dependent protein catabolic process | 6.18439E-05 |
| GO:0032508~DNA duplex unwinding | 6.71827E-05 |
| GO:0032392~DNA geometric change | 6.71827E-05 |
| GO:0051340~regulation of ligase activity | 7.49397E-05 |
| GO:0051726~regulation of cell cycle | 8.50839E-05 |
| GO:0031145~anaphase-promoting complex-dependent proteasomal ubiquitin-dependent protein catabolic process | 0.000171873 |
| GO:0051437~positive regulation of ubiquitin-protein ligase activity during mitotic cell cycle | 0.00022071 |
| GO:0007051~spindle organization | 0.000253783 |
| GO:0051443~positive regulation of ubiquitin-protein ligase activity | 0.000258917 |
| GO:0031396~regulation of protein ubiquitination | 0.000281918 |
| GO:0006974~response to DNA damage stimulus | 0.000294774 |
| GO:0051351~positive regulation of ligase activity | 0.000325775 |
| GO:0051329~interphase of mitotic cell cycle | 0.000338015 |
| GO:0051325~interphase | 0.000402731 |
| GO:0007059~chromosome segregation | 0.000571285 |
| GO:0031398~positive regulation of protein ubiquitination | 0.000693472 |
| GO:0043933~macromolecular complex subunit organization | 0.000696443 |
| GO:0065003~macromolecular complex assembly | 0.000804042 |
| GO:0000075~cell cycle checkpoint | 0.001057154 |
| GO:0000075~cell cycle checkpoint | 0.001057154 |
| GO:0000070~mitotic sister chromatid segregation | 0.001082088 |
| GO:0000819~sister chromatid segregation | 0.001201336 |
| GO:0051436~negative regulation of ubiquitin-protein ligase activity during mitotic cell cycle | 0.001398706 |
| GO:0007017~microtubule-based process | 0.001483476 |
| GO:0051444~negative regulation of ubiquitin-protein ligase activity | 0.001602427 |
| GO:0051352~negative regulation of ligase activity | 0.001602427 |
| GO:0044265~cellular macromolecule catabolic process | 0.002188685 |
| GO:0033554~cellular response to stress | 0.002329694 |
| GO:0031397~negative regulation of protein ubiquitination | 0.002489147 |
| GO:0019941~modification-dependent protein catabolic process | 0.002678633 |
| GO:0043632~modification-dependent macromolecule catabolic process | 0.002678633 |
| GO:0006368~RNA elongation from RNA polymerase II promoter | 0.003176107 |
| GO:0007018~microtubule-based movement | 0.003188069 |
| GO:0006281~DNA repair | 0.003438318 |
| GO:0006354~RNA elongation | 0.003960719 |
| GO:0051603~proteolysis involved in cellular protein catabolic process | 0.004126371 |
| GO:0031570~DNA integrity checkpoint | 0.004248901 |
| GO:0044257~cellular protein catabolic process | 0.00432853 |
| GO:0009057~macromolecule catabolic process | 0.004936279 |
| GO:0006461~protein complex assembly | 0.005140216 |
| GO:0070271~protein complex biogenesis | 0.005140216 |
| GO:0030163~protein catabolic process | 0.00580623 |
| GO:0034621~cellular macromolecular complex subunit organization | 0.005969572 |
| GO:0006302~double-strand break repair | 0.007938979 |
| GO:0006302~double-strand break repair | 0.007938979 |
| GO:0031401~positive regulation of protein modification process | 0.009953105 |
| GO:0000226~microtubule cytoskeleton organization | 0.011227679 |
| GO:0010605~negative regulation of macromolecule metabolic process | 0.01223182 |
| GO:0006351~transcription, DNA-dependent | 0.012409113 |
| GO:0007346~regulation of mitotic cell cycle | 0.013083088 |
| GO:0032774~RNA biosynthetic process | 0.013463055 |
| GO:0070647~protein modification by small protein conjugation or removal | 0.016431795 |
| GO:0006323~DNA packaging | 0.016773933 |
| GO:0006399~tRNA metabolic process | 0.017341455 |
| GO:0031400~negative regulation of protein modification process | 0.017921574 |
| GO:0032268~regulation of cellular protein metabolic process | 0.018023023 |
| GO:0034622~cellular macromolecular complex assembly | 0.020484804 |
| GO:0000724~double-strand break repair via homologous recombination | 0.023848875 |
| GO:0000725~recombinational repair | 0.023848875 |
| GO:0032446~protein modification by small protein conjugation | 0.026653756 |
| GO:0032269~negative regulation of cellular protein metabolic process | 0.027505768 |
| GO:0034660~ncRNA metabolic process | 0.027718373 |
| GO:0000086~G2/M transition of mitotic cell cycle | 0.028808011 |
| GO:0032270~positive regulation of cellular protein metabolic process | 0.02945066 |
| GO:0000079~regulation of cyclin-dependent protein kinase activity | 0.03122232 |
| GO:0051248~negative regulation of protein metabolic process | 0.032237868 |
| GO:0031399~regulation of protein modification process | 0.034939896 |
| GO:0051247~positive regulation of protein metabolic process | 0.035793321 |
| GO:0007126~meiosis | 0.03627307 |
| GO:0051327~M phase of meiotic cell cycle | 0.03627307 |
| GO:0051321~meiotic cell cycle | 0.038626433 |
| GO:0006310~DNA recombination | 0.044884529 |
| GO:0006508~proteolysis | 0.046318161 |
