# Supplementary material for: Network Analysis for the Identification of Differentially Expressed Hub Genes Using Myogenin Knock-down Muscle Satellite Cells
Source: PLoS One. 2015 Jul 22;10(7):e0133597. doi: 10.1371/journal.pone.0133597 (PMC4511796; doi:10.1371/journal.pone.0133597)
Supplement: S7 Table — (DOCX) [file pone.0133597.s007.docx]

**S7 Table.**

**A) Enriched GO terms in cluster 1 detected by MCODE**

| Term | P-value |
| --- | --- |
| Cluster 1 |  |
| NA | NA |
| Cluster 2 |  |
| GO:0050900~leukocyte migration | 0.01738041 |
| GO:0007167~enzyme linked receptor protein signaling pathway | 0.03265612 |
| Cluster 3 |  |
| GO:0044271~nitrogen compound biosynthetic process | 0.01269131 |
| GO:0044271~nitrogen compound biosynthetic process | 0.01269131 |
| GO:0051188~cofactor biosynthetic process | 0.03026081 |
| Cluster 4 |  |
| GO:0007049~cell cycle | 1.1794E-30 |
| GO:0022403~cell cycle phase | 9.6871E-25 |
| GO:0000279~M phase | 4.8774E-22 |
| GO:0022402~cell cycle process | 1.1884E-21 |
| GO:0000278~mitotic cell cycle | 5.1142E-21 |
| GO:0007067~mitosis | 2.1972E-20 |
| GO:0000280~nuclear division | 2.1972E-20 |
| GO:0000087~M phase of mitotic cell cycle | 2.9978E-20 |
| GO:0048285~organelle fission | 4.3845E-20 |
| GO:0051301~cell division | 3.3119E-18 |
| Cluster 5 |  |
| GO:0046822~regulation of nucleocytoplasmic transport | 0.01036559 |
| GO:0032386~regulation of intracellular transport | 0.01525005 |
| GO:0048584~positive regulation of response to stimulus | 0.02451535 |
| GO:0002697~regulation of immune effector process | 0.02949005 |
| GO:0045664~regulation of neuron differentiation | 0.04862972 |
| Cluster 6 |  |
| GO:0016477~cell migration | 0.00152938 |
| GO:0016358~dendrite development | 0.00187932 |
| GO:0051674~localization of cell | 0.00225751 |
| GO:0048870~cell motility | 0.00225751 |
| GO:0006928~cell motion | 0.01056826 |
| GO:0002521~leukocyte differentiation | 0.02413891 |
| GO:0006793~phosphorus metabolic process | 0.03028829 |
| GO:0006796~phosphate metabolic process | 0.03028829 |
| GO:0006468~protein amino acid phosphorylation | 0.0324726 |
| Cluster 7 |  |
| GO:0009611~response to wounding | 0.01913917 |
| GO:0006468~protein amino acid phosphorylation | 0.03484351 |
| GO:0006954~inflammatory response | 0.04916655 |
| Cluster 8 |  |
| NA |  |
| Cluster 9 |  |
| NA |  |

**B) Enriched GO terms in clusters detected by MCL**

| Term | P-value |
| --- | --- |
| GO:0006270~DNA replication initiation | 2.4346E-06 |
| GO:0007049~cell cycle | 2.6773E-06 |
| GO:0007067~mitosis | 8.8241E-06 |
| GO:0000280~nuclear division | 8.8241E-06 |
| GO:0000278~mitotic cell cycle | 9.2536E-06 |
| GO:0000087~M phase of mitotic cell cycle | 1.1412E-05 |
| GO:0048285~organelle fission | 1.5629E-05 |
| GO:0022403~cell cycle phase | 2.3753E-05 |
| GO:0022402~cell cycle process | 2.8609E-05 |
| GO:0000279~M phase | 3.3277E-05 |
